# A rare cause of lower limb deformity

**DOI:** 10.11604/pamj.2018.30.294.16824

**Published:** 2018-08-28

**Authors:** Lieven Dossche

**Affiliations:** 1 Department of Orthopaedic Surgery, Antwerp University Hospital, Antwerp, Belgium

**Keywords:** Congenital deformity, pediatric orthopedics, lower limb, limb dysplasia

## Image in medicine

During an orthopaedic mission in a district hospital in Rwanda, a 10 year old girl was referred for treatment of a bilateral clubfoot. The girl was able to walk. Clinical evaluation (A) showed bilateral marked endotorsion of the lower limb with 90° internal rotation of the foot. There was no equinovarus deformity of the foot with a bilateral plantigrade foot. X-ray evaluation was performed. AP view (B) showed marked bowing and torsion of both tibia and fibula. Lateral lower leg views showed (C) crossing of tibia and fibula with significant diastasis between both bones distally. The diagnosis of congenital diastasis of the inferior tibiofibular joint was made. This is an extremely rare variant of dysplastic tibial anomaly of which the exact etiology is unknown. It is usually associated with equinovarus deformity of the foot and limb shortening. Possible treatments include serial casting if the patient is seen at a very young age, various soft tissue and bony procedures for foot repositioning, Syme amputation and progressive correction with the use of an Ilizarov-external fixator. The girl in the described case had plantigrade feet; given the bilateral presentation, there was no significant leg length discrepancy. Since she was able to walk and local treatment facilities were limited, it was decided to abstain from treatment and accept the deformity.

**Figure 1:**
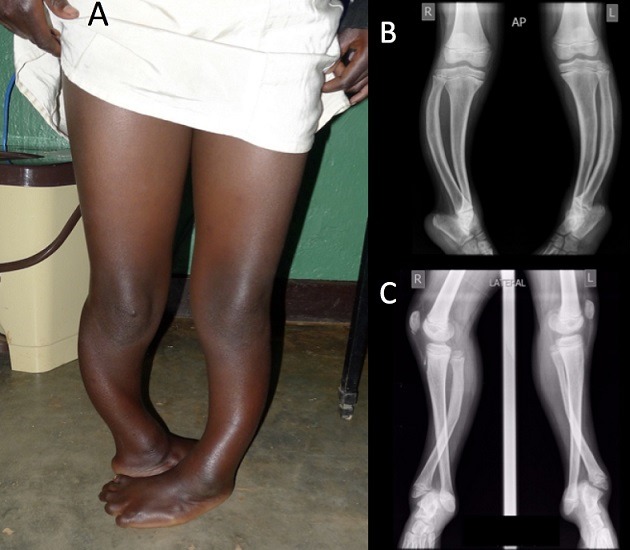
(A) bilateral marked endotorsion of the lower limb with 90° internal rotation of the foot; (B) X-ray evaluation AP view; (C) X-ray evaluation lateral view

